# 3D-Printed Polycaprolactone-Based Containing Calcium Zirconium Silicate: Bioactive Scaffold for Accelerating Bone Regeneration

**DOI:** 10.3390/polym16101389

**Published:** 2024-05-13

**Authors:** Hosein Emadi, Mostafa Baghani, Maryam Masoudi Rad, Bahareh Hoomehr, Majid Baniassadi, Saeid Lotfian

**Affiliations:** 1 School of Mechanical Engineering, College of Engineering, University of Tehran, Tehran 14176-14411, Iran; baghani@ut.ac.ir; 2 Department of Chemical Engineering, Isfahan University of Technology, Isfahan 84156-83111, Iran; m.masoumirad@ch.iut.ac.ir; 3 Department of Materials Engineering, Isfahan University of Technology, Isfahan 84156-83111, Iran; b.hoomehr@ma.iut.ac.ir; 4 Faculty of Engineering, University of Strathclyde, Glasgow G4 0LZ, UK

**Keywords:** calcium zirconium silicate, PCL, fused deposition modelling, cytocompatibility, bioactivity

## Abstract

There is an essential clinical need to develop rapid process scaffolds to repair bone defects. The current research presented the development of calcium zirconium silicate/polycaprolactone for bone tissue engineering utilising melt extrusion-based 3D printing. Calcium zirconium silicate (CZS) nanoparticles were added to polycaprolactone (PCL) porous scaffolds to enhance their biological and mechanical properties, while the resulting properties were studied extensively. No significant difference was found in the melting point of the samples, while the crystallisation temperature points of the samples containing bioceramic increased from 36.1 to 40.2 °C. Thermal degradation commenced around 350 °C for all materials. According to our results, increasing the CZS content from 0 to 40 wt.% (PC40) in porous scaffolds (porosity about 55–62%) improved the compressive strength from 2.8 to 10.9 MPa. Furthermore, apatite formation ability in SBF solution increased significantly by enhancing the CZS percentage. According to MTT test results, the viability of MG63 cells improved remarkably (~29%) in PC40 compared to pure PCL. These findings suggest that a 3D-printed PCL/CZS composite scaffold can be fabricated successfully and shows great potential as an implantable material for bone tissue engineering applications.

## 1. Introduction

Over the past few decades, remarkable progress has been made in the subject of bone tissue engineering, particularly in the development of bone reconstruction, which is at the forefront of this approach [1]. Extensive research has focused on the vulnerability of bone to trauma and fractures in the field of bone tissue regeneration [2]. Bone has complex structures with their own mechanical, chemical, and biological functions [3]. Any missing piece of bone must be replaced with a proper alternative. Today, many methods are used for bone transplantation, including autograft, allograft, xenograft, and substitute bone transplantation [4].

Scaffolds act as a temporary structures, with biocompatibility, strength, osteoconductivity, and sometimes osteoinductivity, that allow for cell proliferation, adhesion, and differentiation, with gradual degradation of the graft being replaced with host cells [5]. Using different methods, scaffolds are formed using a wide array of materials, such as synthetic, biodegradable, and non-biodegradable substances, including polymers, ceramics, metals, and composites. Each offers different properties, such as specific resorption, surface reactivity, and biocompatibility affecting osteoconduction and osteoinduction [6].

Various scaffold fabrication techniques can be applied to form scaffolds, including solvent casting, electrospinning, particular leaching, freeze-drying, melt-moulding, phase separation, and gas foaming [7,8]. However, it is important to state that the majority of these methods have limitations when it comes to creating structures in three dimensions or controlling precise geometrical features. This becomes particularly relevant when considering in vivo conditions, as cells face stress and strain in three dimensions. Therefore, it is highly recommended that 3D structures be utilised in order to achieve the desired outcome. 

Three-dimensional printing technology, also known as the additive manufacturing process, is based on the principle of layered manufacturing and layer-by-layer superposition. Recently, numerous studies have been conducted to utilise 3D printing techniques to facilitate the advancement of a novel category of multifunctional nanocomposites for bone tissue engineering applications [9,10,11]. Depending on the feedstock or the raw material before the printing process, the additive manufacturing techniques can be classified into powder-based 3D printing, selective laser-sintering (SLS), selective laser-melting (SLM), digital light processing (DLP), stereolithography (SL), inkjet printing (IJP), melt extrusion deposition-based three-dimensional printing (MED), etc. [12]. Today, one of the most common processes of additive manufacturing is melt extrusion-based 3D printing, in which the layers build the specimen up from a melted thermoplastic material [13]. The simplicity of the process, reduced costs, and sufficient speed are the significant advantages of extrusion base-melting [14]. 

Due to its significant properties, polycaprolactone (PCL), a part of the polylactone family, has received attention from scientists due to its significance in bone tissue engineering research. This biocompatible polymer’s features are its low degradation rate and less acidic breakdown products compared to other polyesters. The slow degradation of PCL makes it a suitable candidate for bone remodelling, which can be used to control degradation rates [15]. Furthermore, PCL, due to its low melting temperature, a practical polymer for extrusion-based 3D printing [16]. However, pure PCL has low mechanical strength and low bioactivity, while it is expected that it is not osteogenic. Thus, researchers have combined PCL with various bioceramics to improve its properties as a composite scaffold [17,18].

Calcium zirconium silicate (Ca_3_ZrSi_2_O_9_), also known as baghdadite, shows superior bioactivity, cell attachment, proliferation, and biodegradability in comparison to calcium phosphate ceramics, e.g., β-tricalcium phosphate and hydroxyapatite [19,20]. Increased mineral metabolism and ossification are the result of the simultaneous presence of calcium and zirconium in the mentioned ceramic. According to the literature, incorporating elements such as Zr, Zn, and Mg into the network of calcium silicates can control the scaffolds’ mechanical properties and biological performance [21]. Silicon has similar properties to phosphorus in terms of bone formation and growth [22]. Materials based on silicon are important in facilitating bioactivity on surfaces via ion exchange at the interface between the scaffold and tissue, leading to the creation of a layer with a mineral content similar to that of bone [23].

Our previous study [24] evaluated composite scaffolds fabricated with the robocasting method and containing baghdadite nanoparticles and PCL as the matrix. In the current study, the researchers have created PCL/CZS composite scaffolds through the melt extrusion-based 3D printing process. This study aims to comprehensively evaluate the mechanical, thermal, structural, and biological properties of PCL/CZS composite scaffolds for bone tissue engineering.

## 2. Materials and Methods

### 2.1. Materials

Calcium nitrate tetrahydrate and ethanol were obtained from Merck. Other materials used in this study, including polycaprolactone (PCL), zirconium (IV) oxynitrate hydrate, and tetraethyl orthosilicate (TEOS), were all obtained from Sigma-Aldrich (Burlington, MA, USA). PCL has a 1.45 g/mL density and a molecular weight (Mn) of 80,000.

### 2.2. CZS Synthesis

The sol-gel technique was applied to synthesise CZS powder from TEOS (C_2_H_5_O)_4_Si and calcium nitrate tetrahydrate [Ca(NO_3_)_2_·4H_2_O] as described in our previous study [24]. The solution was stirred for 5 h at room temperature (25 °C) and then dried in two steps: one day at 60 °C and two days at 100 °C. The obtained dried gel was then exposed to a 1150 °C calcination process lasting three hours.

### 2.3. PCL/CZS Composites Preparation

To create composite samples of PCL/CZS, the technique of mixing the polymer and powder was employed. In this way, PCL was initially melted. Following this, CZS nanoparticles were introduced into the molten PCL in concentrations of 0, 20, 40, and 60 wt.% and mixed completely to achieve a uniform composite.

### 2.4. 3D Printed Scaffolds Fabrication

At this stage, it is worth mentioning that the sample containing 60 wt.% CZS did not show its printability, and in this way, the samples containing 0 to 40 wt.% were selected for further processing. The samples were labelled based on their respective CZS content (0, 20, and 40 wt.%) as PCL, PC20, and PC40, respectively. The composite materials were fabricated into porous scaffolds using the technique of melt extrusion-based 3D printing system (Chakad, CSS1, Iran, Isfahan). A deposition velocity of 250 mm/min and melting temperature of 80–120 °C was used in this section. The scaffolds exhibited a dimension of 2 × 2 cm^2^, a height of 5 mm, and a pore geometry of 0°/90°.

### 2.5. Scaffolds Characterisations

The surface morphology of the samples in different CZS percentages was carried out using a scanning electron microscope (SEM, Philips XL30, Holland, Amsterdam) at a 20 kV acceleration voltage. All samples were coated with a thin layer of gold before imaging to improve image quality and prevent charging effects. Also, the porosity of the resulting scaffolds was evaluated by Archimedes’ method. 

The phase structure of PCL/CZS composites was analysed using an X-ray diffractometer (XRD, PMD Philips X-Pert) with CuKα radiation = 0.154 nm. The patterns obtained through experimentation were contrasted with the reference patterns developed by the Joint Committee on Powder Diffraction and Standardization (JCDPS) for Ca_3_Si_2_O_4_, specifically CZS (PDF ref. 00-033-0876). The samples were subjected to scanning at a speed of 0.04° per minute, covering a range of 10 to 60 degrees. Utilising the Scherrer method, the size of the crystallites in the CZS nanoparticles was estimated.

(1) $$Dp=\frac{0.94\lambda }{\beta \;Cos\theta }$$

In this equation, λ represents the radiation wavelength, *β* corresponds to the diffraction peak width at half maximum intensity, θ denotes the angle of Bragg diffraction, and *Dp* refers to the average crystallite size. To analyse the size, shape, and distribution of the CZS powder particles, transmission electron microscopy (TEM, Philips CM-200, Holland, Amsterdam) and Image j software (1.52 V) were used.

### 2.6. Mechanical Characterisation of the Scaffolds

In order to assess the mechanical properties, a total of five samples for each group were tested. The researchers performed compression tests using a Hounsfield Materials Testing Machine (Model H25KS, Hounsfield, UK) equipped using a crosshead speed of 1 mm/min and 50% compression strain. The elastic modulus of the specimens was determined by measuring the slope of the stress–strain curves in the elastic region. Compressive stress was calculated at the strain of 50% of each curve. Moreover, the area under the stress–strain curves determined the samples’ toughness and the material’s ability to absorb energy and plastically deform without fracturing.

### 2.7. Thermogravimetric Analysis (TGA)

A thermogravimetric analyser (STA, 449F3, Jupiter, Netzsch GmbH, Selb, Germany) measured the thermal stability of the composite materials. In order to analyse the samples, a heating process was conducted, starting at room temperature and gradually raising the temperature to 600 °C at a rate of 10 °C per minute, with each sample weighing 8–10 mg.

### 2.8. Differential Scanning Calorimetry (DSC)

Differential scanning calorimetry (DSC) measurements were conducted on the composites using a STA 449F3 thermo-gravimetric analyser at a heating rate of 3 °C/min, covering the temperature range from room temperature to 75 °C. Also, the thermal properties of the composites needed to be analysed to evaluate their melting temperature and crystallisation temperature.

### 2.9. In Vitro Bioactivity Evaluation 

Bioactivity testing in vitro, which was conducted by immersing the PCL specimens with 20 wt.% and 40 wt.% CZS in simulated body fluid (SBF), was prepared based on a previous report [25]. The cubic scaffolds, measuring 5 × 5 × 5 mm^3^, were placed in 20 mL of SBF and incubated at 37 °C for a duration of 28 days. The pH of the SBF solution was regularly measured using a pH meter at predetermined time intervals (0–28 days). After the 28-day immersion period, the specimens were taken out of the SBF, gently rinsed with distilled water, and dried at room temperature. To assess the ability to induce apatite formation on the specimen’s surface, SEM and energy-dispersive X-ray spectroscopy (EDS) were utilised. Furthermore, a comparison between the initial and final concentrations of Ca, Si, and P elements was made by analysing the SBF solutions collected after the 28-day immersion using an inductively coupled plasma-optical emission spectrometer (ICP-OES: Perkin Elmer, Optima 7300DV, PerkinElmer, Waltham, MA, USA).

### 2.10. Biodegradation Study

The degradation rate of scaffolds was calculated after placing them in phosphate-buffered saline solution (PBS) with a pH of 7.40. Scaffolds were soaked in 10 mL of PBS solution at 37 °C for 8 weeks. At each time point, samples were monitored for weight loss using the following equation:

(2) $$mass\;loss\%=\frac{\left(w_{0}-w_{d}\right)}{w_{0}}\times 100$$

The initial weight of the scaffolds denoted as $w_{0}$ was measured before the incubation period, while the final weight, represented by $w_{d}$, was determined after the scaffolds had been incubated for a specific duration until they had completely dried.

### 2.11. Cell Viability

The cells’ relative viability was assessed using 3-(4,5-dimethyl thiazolyl-2)-2,5-diphenyl tetrazolium bromide (MTT) obtained from Sigma-Aldrich. At 1, 3, and 7-day intervals, the culture medium was extracted. Both the cell-cultured samples and the control group (with 3 samples in each group) were treated with MTT solution, which consisted of 0.5 g/mL MTT reagent in PBS. After a 4-h incubation period at 37 °C, the dark blue formazan crystals were dissolved in MTT solvent by dimethyl sulfoxide (DMSO) (Bioidea, Iran) and allowed to sit at 37 °C for 30 min. Following this, 100 μL of the dissolved formazan solution from each sample was transferred to a 24-well plate. A microplate reader (Bio Rad, Model 680 Instruments) was used to measure the optical density (OD) of each well at 490 nm wavelength. The relative cell viability was determined using Equation (3) [26]:

(3) $$Relative\;cell\;viability\left(\%\right)=\frac{A_{sample}-A_{b}}{A_{c}-A_{b}}$$

A_Sample_, A_b_, and A_c_ represent the absorbance values of the sample, blank (DMSO), and control (TCP), respectively.

## 3. Results

### 3.1. Morphological Properties

**Figure 1:**
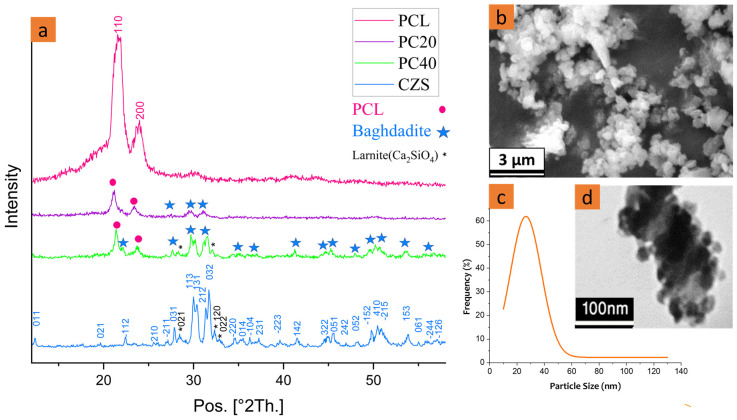
(**a**) XRD pattern of CZS and composites scaffolds, (**b**) SEM, (**c**) nanoparticle size distribution, and (**d**) TEM images of CZS nanoparticles.

The morphology of the synthesised CZS nanoparticles and the XRD pattern of the pure PCL and PCL/CZS composite scaffolds are demonstrated in Figure 1. Scanning electron microscopy (SEM) and transmission electron microscopy (TEM) were used to analyse the shape and size of the synthesised calcium zirconium ceramic. XRD analysis was used to examine the phase structure of the prepared CZS. This confirmed that the CZS phase had formed according to the JCPDS reference pattern 96-901-220. By comparing XRD results, it is evident that the intensity of the CZS peaks increases with the increase in its content in PC20 and PC40 specimens.

**Figure 2:**
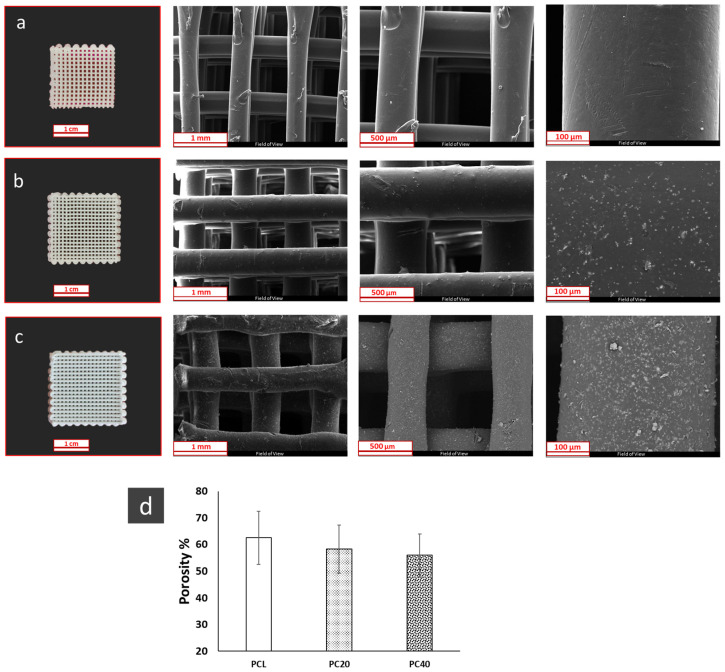
SEM images of PCL and composite scaffolds: (**a**) PCL, (**b**) PC20, (**c**) PC40, and (**d**) their porosity.

The morphology of the pure PCL and composite PCL/CZS scaffolds is represented in Figure 2a–c. The presence of CZS nanoparticles and the increase in their concentration are obvious when the SEM images of three samples are compared, especially in higher magnification images. The change in porosity is represented in Figure 2d. The porosity value is almost constant in all three samples, as it changes in the 55–60% range.

### 3.2. Mechanical Properties of the Scaffolds

**Figure 3:**
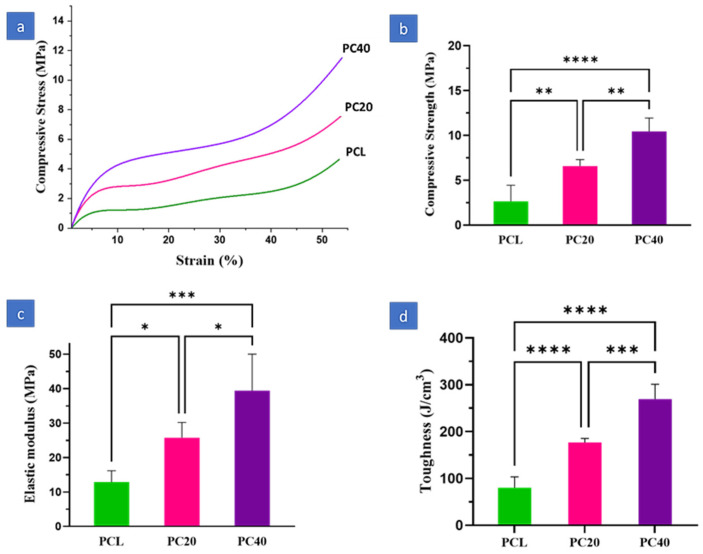
(**a**) Stress–strain curve, (**b**) compressive strength, (**c**) elastic modulus, and (**d**) toughness of scaffolds obtained from mechanical properties (*: *p* < 0.05, **: *p* < 0.01, ***: *p* < 0.001, ****: *p* < 0.0001).

Figure 3 compares the compressive strength, elastic modulus, and toughness of pure PCL and PCL/CZS composite scaffolds. As is evident, the scaffolds’ mechanical characteristics are enhanced by increasing the CZS content. The elastic modulus of PCL, PC20, and PC40 are 12.88 ± 2.96 MPa, 25.76 ± 3.98 MPa, and 39.44 ± 9.46 MPa, respectively. Moreover, the compressive stress of PCL, PC20, and PC40 are 2.64 ± 1.61 MPa, 6.58 ± 0.65 MPa, and 10.34 ± 1.34 MPa, respectively, while the toughness is 80.19 ± 20.81 J/cm^3^, 176.69 ± 7.79 J/cm^3^, and 269.45 ± 28.424 J/cm^3^ for PCL, PC20, and PC40, respectively.

### 3.3. Thermal Properties

During the scaffold fabrication process in the bio-extruder, temperature plays a significant role as one of the processing parameters. Hence, in order to determine the suitable temperatures for the extrusion process, the thermal properties of PCL were investigated along with its composite materials, using DSC and TGA analysis. The composition of the 3D-printed scaffolds and the quantity of PCL and CZS incorporated within them were assessed using thermogravimetric analysis by measuring mass loss at 25–600 °C.

**Figure 4:**
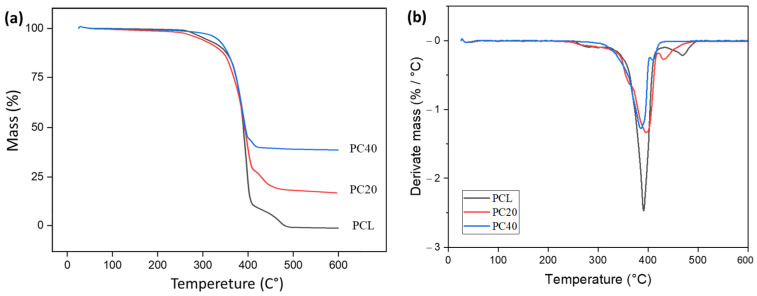
(**a**) Thermogravimetric curves and (**b**) first derivative of TGA in PCL-based scaffolds.

Figure 4 displays the TGA and DTG curves. Figure 4a demonstrates the mass loss of samples after exposure to heat. As can be seen, the PCL sample had the maximum degradation, while the PC40 sample had the maximum mass remaining. Figure 4b shows the mass loss derivation for samples after heat exposure. The first derivative of TGA helps identify the temperature range with the most significant mass loss.

Table 1 indicates the predicted thermal parameter values from TGA analysis. The parameters in this table are mass loss percentages at 150 and 600 °C, remaining mass percentages at 150 and 600 °C, and inflection points (temperatures at the samples’ DTG peaks).

**Figure 5:**
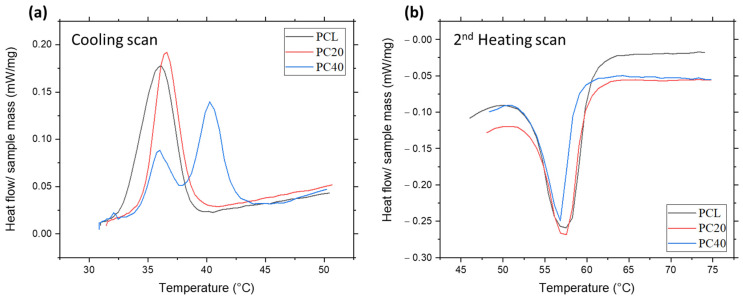
(**a**) DSC cooling and (**b**) second heating curves of PCL/CZS nanocomposite scaffolds.

Figure 5 displays the DSC curves for the cooling and the second heating phases of the PCL and PCL/CZS composites. In Figure 5a, the cooling scan analysis of the samples can be observed. In this figure, the peaks indicate the crystalline regions. In the second heating scan (Figure 5b), the melting range of the samples can be recognised. Two distinct thermal effects can be detected in all the DSC curves: melting temperatures across 55–60 °C and crystallisation at around 35–40 °C. It can be observed that the melting temperature remains consistent between 55 °C and 60 °C for all materials, regardless of the inclusion of ceramic filler.

Table 2 indicates the predicted thermal parameter values. Parameter values in this table are, respectively, onset crystallisation temperature (T_C_ onset), crystallisation temperature (T_C_), onset melting temperature (T_m_ onset), melting temperature (T_m_), and melting and crystallisation enthalpies (${\Delta H}_{m}$ and ${\Delta H}_{c}$) obtained from the DSC peak analysis. 

### 3.4. Biological Properties of the Scaffolds

**Figure 6:**
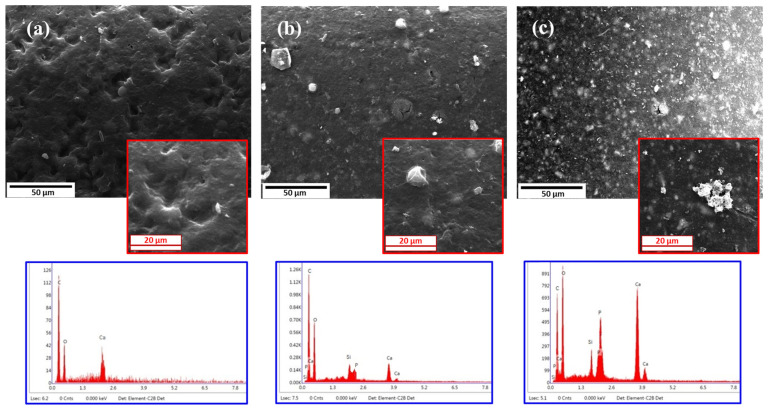
EDS analysis of the PCL and composite scaffolds after 28 days of immersion in SBF solution: (**a**) PCL, (**b**) PC20, (**c**) PC40.

Figure 6 shows the surface of the scaffolds and the energy-dispersive X-ray spectroscopy (EDS) of the deposits formed on their surface after 28 days of immersion in simulated body fluid (SBF) solution. 

Table 3 also shows the weight percent distribution of the elemental composition of the precipitates on the surface scaffold based on EDS analysis. It can be seen that the number of deposits increases by enhancing the CZS content from 0% in pure PCL to 40% in the PC40 specimen. The Ca and P peaks sharpen in EDS results as the amount of CZS increases in the samples.

**Figure 7:**
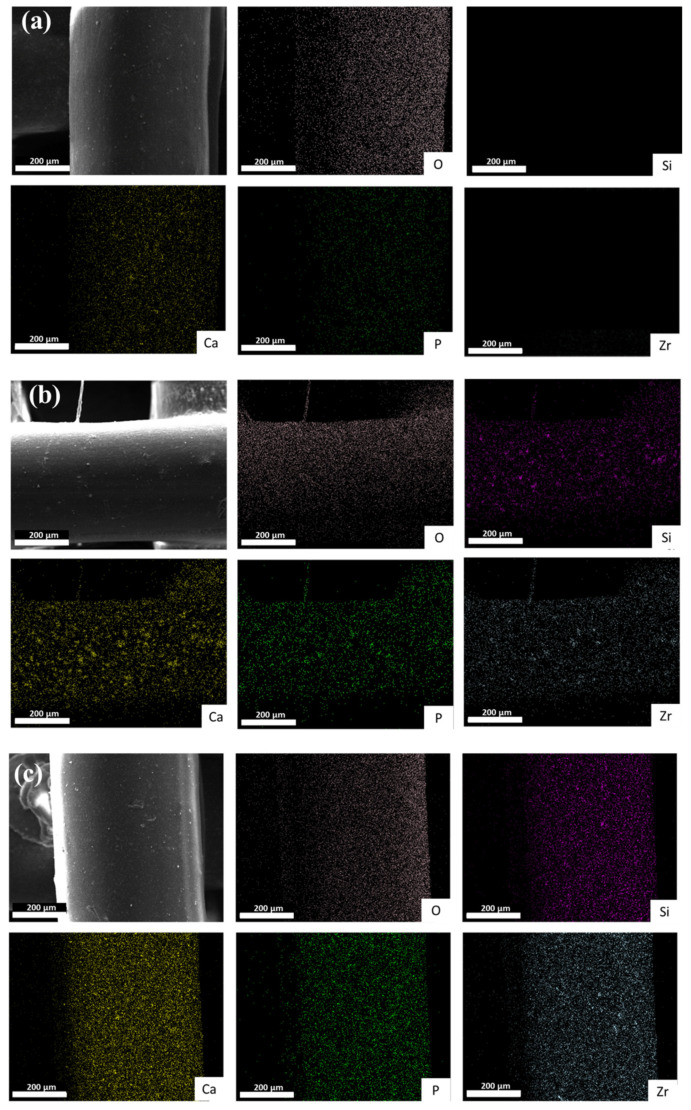
Map analysis of (**a**) PCL, (**b**) PC20, and (**c**) PC40 scaffolds after 28 days of immersion in SBF solution.

Figure 7 represents the EDS map of the pure PCL and PCL/CZS composite scaffolds after 28 days of immersion in SBF solution. According to Figure 7a–c, the concentration of Ca and P increases by increasing the CZS content in the scaffolds, representing the formation of Ca-P deposits. Furthermore, the Si and Zr contents increase remarkably due to the higher amount of CZS in composite scaffolds.

**Figure 8:**
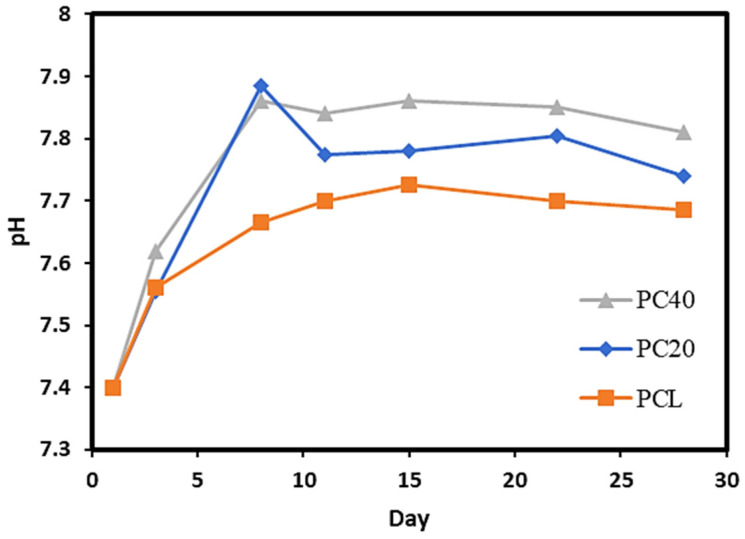
pH evaluation of PCL and composite scaffolds after 28 days’ immersion in SBF solution.

Figure 8 demonstrates the pH variation in pure PCL and PCL/CZS scaffolds after 30 days of immersion in SBF solution. The increase in pH after 7 days of immersion is higher in the PC40 specimen compared to pure PCL and PC20 due to the higher CZS content and, hence, higher Ca ion release in the SBF.

Table 4 compares the concentration of Ca, P, and Si ions in the SBF solution after 28 days of immersion to their original concentration (SBF is used as a control sample). The PC40 specimen experienced the highest decrease in Ca and P concentration, which confirms the SEM results, which show higher Ca-P deposit formation on the PC40 specimen.

### 3.5. Biodegradation Evaluation

**Figure 9:**
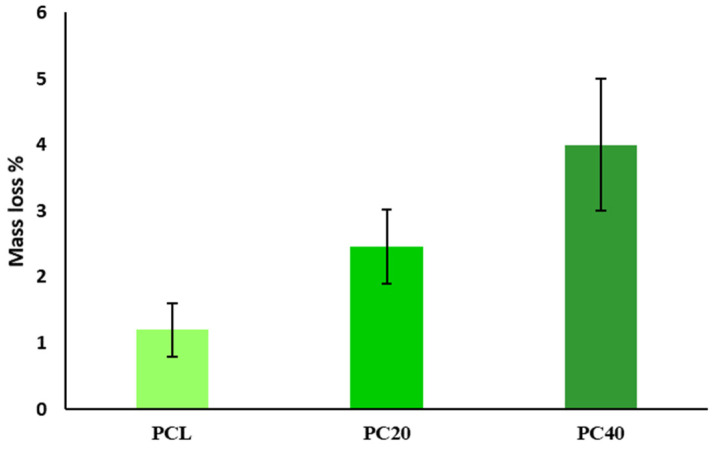
Mass loss percentages of scaffolds after 8 weeks’ immersion in PBS solution.

Figure 9 compares the mass loss percentage in pure PCL and PCL/CZS composites after 28 days of immersion in phosphate buffered saline (PBS) solution. According to the results, the degree of degradation increases remarkably with the increase of the CZS content from 0 wt.% to 40 wt.%. The concentration of Si and Ca ions in PBS solution after 21, 42, and 70 days of immersion is represented in Table 5. According to these data, the amount of Ca decreased with time, while the Si ion content increased within this period.

### 3.6. Cell Viability 

**Figure 10:**
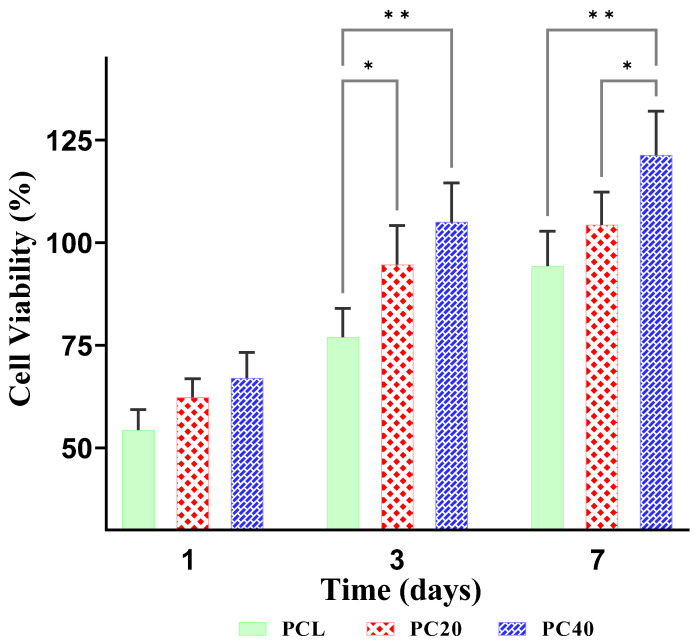
The relative viability of MG63 cells cultured on different specimens after 1, 3, and 7 days of culture. (*: *p* < 0.05, **: *p* < 0.01).

Figure 10 represents the relative MG63 cell viability in pure PCL and PCL/CZS specimens after 1, 3, and 7 days of culture. According to these results, the PC40 sample shows significantly higher viability after 3 and 7 days, which can be related to the higher CZS content.

## 4. Discussion

### 4.1. Morphological Properties

Figure 1 shows TEM and SEM images of CZS powders and XRD patterns of CZS and PCL/CZS composites. According to SEM and TEM images (Figure 1b,d), CZS nanoparticles possess semi-spherical morphology. Due to the fact that the sphere has the minimum surface area among all geometric shapes, material tendencies naturally favour spherical shapes to ensure the lowest possible state of energy. However, due to the nanostructure’s high surface energy and the agglomeration of nanoparticles, the shape created by placing particles next to each other is not completely spherical, so the images showed that the CZS powder consisted of semi-spherical particles. Previously, Pahlevanzadeh et al. [27] observed semi-spherical shapes in nanobaghdadite powder particles. The diagram of the CZS particle size distribution based on TEM images is represented in Figure 1c. The results show that about 60% of the particles were in the range of 20–40 nm, and the average particle size is 26.5 ± 13 nm. The crystalline size of synthesised CZS powder calculated by the modified Scherrer equation (Equation (1)) was about 33 ± 7 nm, which was in good agreement with the particle size observed in the TEM image.

Figure 1a demonstrates the XRD patterns of the CZS, pure PCL, PC20, and PC40 scaffolds. The noises observed in the XRD pattern of the scaffolds are due to their porous nature. The CZS peaks were more detectable in the PC40 specimen as it contains higher CZS values. In contrast, the PC20 pattern is more inclined towards polycaprolactone peaks.

In the XRD patterns, two peaks were characterised by PCL at 2θ = 21.7° and 2θ = 23.9°. The intensity of the PCL peaks was different in various samples as a result of crystallinity alteration after composite extrusion. According to the literature, PCL-hydroxyapatite composites have shown a similar pattern before, where an increase in the HA content caused a decrease in the intensity of the polymer peaks [28,29].

According to JCPDS reference pattern 96-901-2206, the predominant detected phase is baghdadite with the chemical formula of Ca_12_Zr_4_Si_8_O_36_, which has a monoclinic structure. Besides baghdadite, new and tiny peaks were detected and indexed as dicalcium silicate phase (Ca_2_SiO_4_ with monoclinic structure), also known as larnite, at approximately 2θ = 28.10, 32.17, and 32.78, according to JCPDS reference pattern 96-901-2793. According to the literature [30], at the temperature of 925 °C, the formation of calcium silicate, larnite 2CaO·SiO_2_, begins. As the sintering temperature was 1150 °C in the present study, the formation of this phase is explained. Sadreddini et al. [31] also synthesised baghdadite particles and observed larnite as an impurity in their XRD pattern. They attributed the formation of this impurity to the fast reaction of Si with Ca, even before the presence of Zr, and the creation of the Ca–Si phases. 

The morphology of scaffolds incorporating different concentrations of CZS nanoparticles is depicted in Figure 2. SEM images clearly demonstrate the scaffold’s porous structure. The CZS particles were found to be dispersed homogeneously throughout the polymer matrix. However, with the increase in the CZS content in the scaffolds to more than 40 wt.%, particles tended to form agglomerates and cover the surface of the scaffolds, and the ink produced was no longer printable. One major benefit of 3D-printed scaffolds is the presence of interconnected pores. As tissue engineering scaffolds, such structures can mimic the natural extracellular matrix (ECM) well. The scaffolds produced in this study demonstrate a significant level of porosity and interconnected pore morphology, as presented in Figure 2. This enables efficient oxygen and nutrient transfer in the scaffolds, leading to improved cell migration and regulation of ECM formation [32]. The bone regeneration process begins when cells and nutrients penetrate the scaffold through interconnected pores, and osteoblast cells begin growing on the scaffold’s surface. Besides improving nutrient and oxygen transfer into the inner pores, high porosity and pore interconnectivity are also effective at removing metabolic waste products [33,34]. The porosity of scaffolds can be seen in Figure 2d. An appropriate porous scaffold with interconnected pores provides an environment for promotion of cell infiltration, migration, and vascularisation, facilitating the flow of nutrients and oxygen and waste removal while maintaining mechanical stresses [35]. In this study, the results show that the porosity of scaffolds is in the range of 50–60%, and there is no significant difference in the porosity of PCL, PC20, and PC40 scaffolds.

### 4.2. Mechanical Properties 

Figure 3 demonstrates the influence of CZS inclusion on the mechanical properties of the PCL-based scaffolds containing various CZS concentrations (0 wt.%, 20 wt.%, and 40 wt.%). According to Figure 3, Young’s modulus, compressive stress, and toughness were increased by adding CZS nanoparticles. The bulk properties of PC40 scaffolds increased more than three times compared to the PCL scaffolds. The inclusion of CZS particles in the PCL matrix improved the strength and stiffness of scaffolds. For example, based on Figure 3d, the toughness of PC20 and PC40 is about 2.2 and 3.3 times higher than that of the PCL scaffold. This issue could be caused by the fact that CZS has increased strength in the composite structure, which is also in line with the law of mixtures, where the combination of constituent materials determines the composite properties, following the mixture rule.

Figure 3b shows a notable increase in compressive strength, from 2.64 MPa to 6.58 MPa and then 10.34 MPa when the CZS content increased from 0 wt.% to 20 and 40 wt.%. Other studies have confirmed that adding ceramic to the scaffold increases its mechanical properties [36,37,38]. According to Sadeghzade et al. [39], the addition of CZS to hardystonite significantly increased the compressive modulus and strength.

The scaffold’s mechanical properties should be in line with those of the surrounding tissue in the implantation site. Consequently, cells must not be subjected to excessive compressive or tensile loading because they may affect the physiological conditions, which will then not be properly functionalised [40]. One of the key challenges in regenerating different tissues, especially bone, is manufacturing scaffolds with appropriate mechanical properties [41]. The compressive strength of human cancellous bone is about 4–50 MPa, and tensile values range from 5 to 40 MPa, according to various studies [42,43,44]. This study’s compressive test results indicated PCL/CZS scaffolds had mechanical properties similar to those of bone; therefore, they can be considered for bone regeneration applications. As a result, the specimen containing 40 wt.% CZS would have the highest strength in healing injured bone.

### 4.3. Thermal Properties

Figure 4 displays the TGA and DTG curves. The behaviour of thermal decomposition can be categorised into three separate regions. It can be observed in Table 1 and Figure 4 that the mass loss is minimal, reaching a maximum of only one percent, up to 150 °C. In these areas, removing water and moisture from the scaffold structure (including absorbed and bound water) leads to partial mass loss.

However, with the addition of CZS at 150 °C, PC20’s mass loss is three times higher than PCL’s, and PC40’s mass loss is twice as high as PCL’s. The issue can be attributed to PCL’s hydrophobicity. By introducing CZS, the hydrophobicity of the scaffolds decreases, leading to higher water absorption. Also, the addition of inorganic fillers such as CZS can decompose some polymer chains [45]. High temperatures weaken and break bonds between polymer chains, forming smaller molecules [46]. Also, in the presence of oxidising agents such as oxygen, this process can speed up, resulting in exacerbated degradation [47].

No significant mass loss was detected at room temperature to 360 °C. Tran and Trakoolwannachai et al. [25,39] reported that this finding demonstrated the PCL surface’s hydrophobic nature. The thermograms exhibit a sharp decrease in sample mass occurring at degradation temperatures ranging from 360 to 430 °C (Figure 4). This reduction corresponds to the polymer’s structural decomposition. No significant mass loss was seen over 430 °C. The mass of all samples remained relatively constant after reaching a temperature of 475 °C, and this mass was directly proportional to the amount of CZS present in the samples (Figure 4b).

Scaffold mass losses at 600 °C were 99.1%, 80.7% and 61.3% in PCL, PC20, and PC40, respectively. Most likely, these values are slightly lower than the targets of 20 and 40 wt.% due to minor moisture content and organic composition of the PCL, especially in bio-CZS powders, which are estimated to range between 1 and 4 wt.% in scaffolding structure. This thermal degradation behaviour has been observed previously in 3D printed bio-composites such as PCL/hydroxyapatite scaffolds [48,49]. The explanation is the release of water that has been absorbed from the surface of CZS, constituting approximately 5% of the partial weight of the particle’s total weight. It is evident from the results that a pure PCL scaffold has no residual material at 600 °C, suggesting that this scaffold consisted of organic components and had completely decomposed at this temperature. Researchers have previously observed this similar thermal behaviour of polycaprolactone [49,50,51].

Figure 5 displays the DSC curves for the cooling and the second heating phases of PCL and PCL/CZS composites. Table 2 displays the anticipated values of the thermal parameters. Both the composite and the pure PCL melted at around the same temperature (55–60 °C). In another study, results indicated almost the same melting temperature range for 3D-printed scaffolds containing PCL [48]. According to Figure 5, the melt crystallisation exotherm was observed in the pure PCL, PC20, and PC40 composites. The onset of PCL crystallisation occurred at 37.3 °C during cooling after the melting process. The melt crystallisation temperature for both composites was marginally greater than that for pure PCL scaffolds. Based on these results, it seems that CZS content influences nucleating activity. This effect has also previously been observed in hydroxyapatite PCL L-lysine composites [50]. Endothermic melting peaks are visible in DSC curves when the samples are heated for a second time. The findings indicate that the melting point of pure PCL is 57.5 °C, which is about 0.7 °C higher than the melting point of composite scaffolds. 

The melting point of PCL is typically around 55 to 60 °C [52,53,54]. Regardless of the inclusion of ceramic filler, the melting temperature remains consistent between 55 and 60 °C for all scaffolds. Similar results were observed for PCL/diatomaceous earth nanocomposites [55]. Also, in their study, the addition of diatomaceous earth within the PCL matrix slightly decreased T_m_ (by up to 4 °C), which agrees with the results obtained in this research.

The melting and crystallisation enthalpies from the integration of DSC peaks are shown in Table 2. As is known, melting enthalpy decreases from 31.15 to 20.79 J/g, and crystallisation enthalpies decrease from 21.45 to 12.47 J/g in PC40 compared to PCL with the addition of CZS. 

Several studies have shown that fillers within the polymeric matrix can decrease ΔH, indicating a decrease in polymer crystallinity, as reported in the literature [56,57]. According to the obtained results, the crystallinity of the samples decreases with the increase of CZS. This issue leads to an increase in the rate of degradation of scaffolds in aquatic environments, which is consistent with the results obtained in the biodegradability assessment in Section 3.5. 

The present study shows that the PCL/CZS composite’s melting endotherm peaks reached a maximum of 61.4 °C. Considering that thermal degradation commences around 300 °C for all materials, as revealed by TGA analyses, this implies that no deterioration is expected when 3D printing is carried out within the 80–120 °C range.

### 4.4. Bioactivity Assessment

Figure 6 represents apatite formation on the surface of the PCL, PC20, and PC40 specimens after 28 days of immersion in SBF solution. As is obvious, by increasing the CZS content in the composite, the number of apatite nuclei is enhanced significantly. Previously, nanocomposite films that contained CZS particles showed an outstanding apatite layer on their surface and more bioactivity than pure polymer films when subjected to a simulated body solution for 28 days [58].

The EDS test results represented in Table 3 show how the elements are spread out on the scaffold surface. It is shown that the surface of composite scaffolds has more calcium and phosphorus than the surface of PCL scaffolds, and it provides a favourable environment for the development and germination of apatite compounds suitable for bone regeneration. The decrease in the percentage of carbon in PC20 and PC40, in addition to the decrease in the percentage of polymer chains in the scaffold structure, indicates an increase in the formation of apatite on the scaffold surface, which leads to a decrease in carbon on the composite scaffolds.

The incorporation of CZS in the structure of PC20 and PC40 scaffolds leads to the presence of silicon and zirconium elements, causing the release of ion exchange with the SBF solution. By immersing the specimen containing CZS in SBF solution, the first reaction that takes place is ion exchange with the solution. In this way, Ca^2+^, Zr^4+^, and Si^4+^ dissolve in SBF, and ion exchange happens when H+ and H3O+ are present in the solution, which results in pH enhancement. Increased pH leads to silica bond breakup and reformation of silanol groups on the surface of composite specimens containing CZS. The next step consists of the formation of calcium phosphate groups, which results in a reduction in pH. By diffusing carbonate present in SBF into the calcium-phosphate deposits, crystallisation occurs and hydroxy carbonate apatite forms [59].

According to Figure 7**,** which demonstrates the EDS map and hence the distribution of Si, O, Zr, P, and Ca elements in the PCL, PC20, and PC40 specimens, it is obvious that the surface of PC40 is enriched with calcium-phosphorous deposits significantly higher than those of the two other specimens, which is related to the higher content of calcium in this specimen, which dissolves in SBF solution and redeposits as calcium-phosphorous nuclei.

Figure 8 illustrates pH changes in the SBF solution caused by 28 days of immersion with the PCL, PC20, and PC40 specimens. According to this figure, the pH value in all specimens experiences an increase and then gradually stabilises. By immersing the scaffolds in SBF solution, PCL hydrolysis starts on the scaffolds’ surface because of the PCL’s low hydrophilicity. However, in PC20 and PC40 specimens that contain CZS, the hydrolysis process occurs simultaneously on the surface and in the interior of the scaffolds, resulting in the release of Si, Ca, and Zr ions into the SBF solution. 

The degradation of the polymeric part of the scaffolds (PCL) leads to a decrease in pH due to the acidic degradation products, which is an autocatalytic phenomenon, as these acidic products remain inside the scaffold and accelerate the degradation and pH decrease [60]. By incorporating CZS into the scaffold matrix, the release of Si and Ca ions compensates for the reduction of pH due to the acidic degradation of PCL. Therefore, the pH of SBF experiences a higher increase compared to the PCL specimen. 

The results of the ICP test, which shows the variation in Ca, P, and Si ion concentrations over a period of 28 days of immersion in SBF solution, are represented in Table 4. According to Table 4, the concentration of Ca and P ions decreased in all specimens after 28 days of immersion, confirming the formation of calcium-phosphate deposits. After immersing the specimens in the SBF solution, the Ca-P crystals re-precipitate onto the surface of the scaffolds due to the supersaturation of Ca^2+^ and P^5+^ ions.

When the Si-O-Si network undergoes dissolution, it leads to the creation of silanol bonds. These bonds then act as a substrate for the deposition of apatite when both Ca and P are present. However, due to the higher apatite-forming ability of the PC40 specimen compared to the other specimens with lower or no CZS content, the concentration of Ca and P ions is lower in the SBF solution, which is in agreement with the results obtained from the EDS map and FESEM images which represented higher apatite formation in PC40 specimen. The higher Si content in the PC40 specimen is due to higher CZS concentration in this specimen and, hence, higher Si release into the SBF solution.

### 4.5. Biodegradation Evaluation

It is imperative for a scaffold to be biodegradable in order to facilitate the replacement of the implanted structure by body tissues, allowing for cell growth and eventual replacement of the scaffold. Additionally, the implant must be non-toxic and easily degradable without causing any disruption to surrounding tissues or organs. To achieve degradation while simultaneously promoting tissue formation, there needs to be a regulated transport of cells, such as macrophages, with controlled inflammatory responses [61]. Figure 9 represents the percentage of mass loss in PCL, PC20, and PC40 scaffolds after 8 weeks of immersion in PBS solution. As shown, the mass loss percentage in the PC40 specimen is approximately 6% and a great deal higher than in other specimens (PCL~1% and PC20~2.5%). 

According to a study by Sung et al. [62], polymers with a fast degradation rate, such as PLGA, can lead to a decrease in pH due to the hydrolysis of the ester bond into acidic monomers. This also happens for PCL polymers. However, as the degradation rate of PCL is significantly lower than that of PLGA (as PCL has higher molecular weight and hydrophobicity), the pH of the environment is less detrimental to cell proliferation. Consequently, the rate of degradation can affect cellular interactions, including cell proliferation, tissue synthesis, and host response [63]. In the present study, part of the PCL polymer was replaced by CZS, which resulted in higher degradation and compensation for the probable adverse effect of PCL and improved apatite formation and cell viability, which will be discussed further in the next section.

Scaffold degradation typically involves two main mechanisms: bulk degradation and surface erosion. The degradation process commonly consists of a blend of these two methods, with varying proportions based on the material composition. Surface erosion takes place at the scaffold’s interface with the external environment, progressing from the outer layer towards the inner core, whereas bulk degradation occurs uniformly throughout the scaffold’s entire volume [61]. The degradation of biopolymers involves breaking atomic bonds, resulting in the formation of oligomers, monomers, or other species with low molecular weight. The hydrolytic breakdown of PCL leads to the formation of carboxylic acid terminal groups. These groups lower the pH in the vicinity of the scaffold, which in turn negatively affects bone response. Additionally, this process can cause an excessive depletion of mineral and organic salts from bone tissue, potentially resulting in cytotoxic effects. Hence, adding CZS and reducing PCL content is advantageous in controlling the pH variation in the same conditions as the body’s environment. Bioceramics undergo biodegradation through various mechanisms, such as hydrolytic decomposition, degradation mediated by cells, and fragmentation into smaller particles due to mechanical stresses and scaffold deterioration [61].

In Section 3.1, it was demonstrated that by increasing CZS content, porosity percentage decreased. According to a review by Tajvar et al. [61], in certain polymeric scaffolds like PCL or PLGA, a reduction in porosity can result in increased degradation caused by the autocatalytic impact of acidic degradation products. This phenomenon can be attributed to the accumulation of degradation products, which amplifies their autocatalytic effect. This can explain the lower degradation rate of PCL specimen despite its higher porosity. However, biodegradable bioceramics such as CZS are more effective in the degradation field than the porosity percentage.

Table 5 represents the variation in Ca and Si ions in PBS solution after 70 days of immersion for all specimens. In all specimens, the number of Ca ions present in the PBS solution decreases with the passage of time, while the Si content increases due to lack of Si consumption. The decrease in the content of Ca ions in PBS solution by increasing the CZS is due to the precipitation of Ca deposits on the surface of scaffolds. Silicon ion release can signify the level of degradation and decomposition of the scaffolds since silicon is not naturally found in the PBS solution.

### 4.6. Cell Viability

CZS exhibits extraordinary biological characteristics along with its physical and mechanical attributes. In contrast to pure wollastonite, CZS demonstrates enhanced degradability, resulting in a more stable structure that is highly suitable for cell culture and the treatment of extensive bone defects [59]. According to the literature [64], the stability of pH around 7.2–7.4 is a vital factor for cell metabolism and hence for cell proliferation. Hoomehr et al. [64] reported that the presence of zirconia controls the Ca ion release from bioactive glass and develops a stable pH in the surrounding medium, which favours cell proliferation. As stated by Sadeghzade et al. [59], the presence of Si, Ca, Mg, and Zr in CZS and diopside play vital roles in bone formation, regeneration and remodelling in vivo. Furthermore, the release of these elements, due to the degradation of the mentioned ceramics, results in differentiation and proliferation of osteoblast cells in vitro. Figure 10 demonstrates the variation in cell viability in PCL, PC20, and PC40 specimens after seeding MG63 cells from day 1 to day 7. According to Figure 10, the viability of the cells after 1, 3, and 7 days is significantly higher in the PC40 specimen compared to the two other groups.

The PC40 specimen exhibited a remarkable increase in cell viability (121 ± 10% (control)) after 7 days of culture, surpassing the PC20 (104% (control)) and PCL (94% (control)) specimens. A notable observation was made regarding the MG63 cells, as they exhibited a substantial improvement in proliferation when cultured on the PC40 specimen, rising from 67 ± 6% (control) on day 1 to 121 ± 10% (control) on day 7 by a significant margin (80.6%). In comparison, the data collected over a period of 7 days revealed that PC20 and PCL had much lower proliferation rates. According to the results reported in Figure 9, the improvement in cell viability in the PC40 specimen compared to PCL (28.7%) after 7 days of culture is remarkably higher than that in the PC20 specimen compared to the same group (10.6%). These observations can be related to the higher release of Si and Ca ions from the PC40. According to the literature [65,66,67], the release of Si element results in higher differentiation and proliferation of osteoblast cells.

## 5. Conclusions

This study aims to expand 3D-printed polycaprolactone-based scaffolding containing calcium zirconium silicate for accelerating bone regeneration. By using melt extrusion-based 3D printing, PCL/CZS scaffolds with various CZS percentages were produced. The fabricated scaffolds were compared in terms of thermal, mechanical, and morphological properties and in vitro bioactivity. SEM images revealed the presence of 3D scaffolds with open and interconnected pores. PC40 exhibited a significantly higher Young’s modulus and compressive strength, approximately 3 and 4 times greater, respectively, than those of the PCL reference sample. Moreover, it had a porosity level exceeding 55%. The behaviour of thermal decomposition was assessed, and the thermal stability of 3D-printed scaffolds was shown, while TGA analysis indicates that mass loss remained dependent on the quantity of CZS present in the sample. Increasing the CZS content significantly enhanced the bioactivity of the scaffolds. Degradability testing indicated that a higher concentration of CZS nanoparticles led to a greater decomposition of silicon-containing groups, resulting in a faster degradation rate for the composite scaffolds in comparison to the PCL scaffold. Furthermore, the cell viability improved remarkably (29%) in 40 wt.% CZS compared to the pure PCL. In summary, the combination of a porous structure, excellent biological responses and great mechanical properties makes PCL/CZS scaffolds highly promising for use in bone tissue engineering.

## Figures and Tables

**Table 1:** Thermal parameters from TGA analysis of PCL-based porous scaffold.

Sample	Mass Loss at 150 °C (%)	Remained Mass at 150 °C (%)	Mass Loss at 600 °C (%)	Remained Mass at 600 °C (%)	Inflection Point (°C)
PCL	0.3	99.7	99.1	0.9	390.5
PC20	1.0	99.0	80.7	19.3	394.9
PC40	0.6	99.4	61.3	38.7	386.6

**Table 2:** Crystallisation and melting temperature parameters of PCL-based porous scaffold.

Sample	T_c_ Onset (°C)	T_c_ (°C)	${∆H}_{c}$ (J/g)	T_m_ Onset (°C)	T_m_ (°C)	${∆H}_{m}$ (J/g)
PCL	38.2	36.1	31.15	54.0	57.5	−21.45
PB20	38.6	36.6	24.62	54.0	57.5	−16.26
PB40	41.5	40.2	20.79	54.0	56.8	−12.47

**Table 3:** Specific element content values results of the EDS analysis.

	Sample	PCL	PC20	PC40
Element (Weight %)				
C		54.5	33.1	19.5
O		41.3	31.2	23.1
Ca		2.7	14.9	24.3
P		1.5	7.5	13.3
Zr		-	7.1	10.1
Si		-	6.2	9.7

**Table 4:** Ion concentration of Ca, P, and Si elements in SBF solution after 28 days of immersion.

	Specimen	SBF	PCL	PC20	PC40
Ion Con. (mg/L)					
Ca		104	80.1	63.3	59.9
P		35	27.2	19.3	14.2
Si		0	0	1.2	3.3

**Table 5:** Ion concentration of Ca and Si elements in PBS solution at different times.

Specimen	PCL (mg/L)		PC20 (mg/L)		PC40 (mg/L)	
Time	Ca	Si	Ca	Si	Ca	Si
Day1	-	-	-	-	-	-
Day21	-	-	5.3	0	3.8	1.3
Day42	-	-	4.8	1	3.3	4.0
Day70	-	-	3.5	2.2	2.9	14.8

## Data Availability

The data presented in this study are available on request from the corresponding author. The data is not publicly available because it is part of an ongoing study.
